# Interneuron Types and Their Circuits in the Basolateral Amygdala

**DOI:** 10.3389/fncir.2021.687257

**Published:** 2021-06-10

**Authors:** Norbert Hájos

**Affiliations:** Laboratory of Network Neurophysiology, ELRN Institute of Experimental Medicine, Budapest, Hungary

**Keywords:** inhibitory, microcircuits, wiring principles, GABAergic, rat, mice

## Abstract

The basolateral amygdala (BLA) is a cortical structure based on its cell types, connectivity features, and developmental characteristics. This part of the amygdala is considered to be the main entry site of processed and multisensory information delivered *via* cortical and thalamic afferents. Although GABAergic inhibitory cells in the BLA comprise only 20% of the entire neuronal population, they provide essential control over proper network operation. Previous studies have uncovered that GABAergic cells in the basolateral amygdala are as diverse as those present in other cortical regions, including the hippocampus and neocortex. To understand the role of inhibitory cells in various amygdala functions, we need to reveal the connectivity and input-output features of the different types of GABAergic cells. Here, I review the recent achievements in uncovering the diversity of GABAergic cells in the basolateral amygdala with a specific focus on the microcircuit organization of these inhibitory cells.

## Introduction to The Connectivity of The Basolateral Amygdala

Amygdala is the brain region where at least 13 different nuclei are defined with typical neuron types, developmental origin, and connectivity patterns (Pitkanen et al., 1997; Swanson, 2003), playing a role in surprisingly diverse functions, including aversive memory formation, decision-making, social interactions, affective and parental behavior, and homeostatic control, just to list a few (LeDoux, 2000; Phelps et al., 2014). Two different amygdala parts, the lateral (LA) and basal (BA) nuclei, which are often referred to as the basolateral amygdala (BLA), are among the most studied areas. Notably, both nuclei can be further divided into distinct subnuclei (Pitkanen et al., 1997; Swanson and Petrovich, 1998), the functions of which have recently begun to be uncovered using subnucleus-specific manipulations of neural operation (Kim et al., 2016). In spite of a separate role for LA and BA in certain cognitive processes (Janak and Tye, 2015; Manassero et al., 2018), there is no evidence to date of the presence of different cell types or distinct wiring principles in these two amygdalar nuclei. Therefore, I will review the microcircuit organization of the basolateral amygdala as a whole.

Based on the cell types, their connectivity features, and developmental characteristics, the BLA is a cortical structure. Accordingly, glutamatergic excitatory projection cells expressing vesicular glutamate transporter type 1 (VGluT1; Andrasi et al., 2017) are the most numerous neurons in this amygdala region (80–85%; Vereczki et al., 2021). The dendrites of the principal cells (PC) are densely decorated with spines and their axon arborizes within the nucleus, giving rise to local collaterals, but they also project to other amygdala regions and remote cortical and/or subcortical areas (Sah et al., 2003). The BLA, similarly to all cortical structures, is connected to the thalamus and basal ganglia in addition to other cortical networks (McDonald, 1991; Turner and Herkenham, 1991; Sah et al., 2003). VGluT1-expressing glutamatergic axonal varicosities (Fremeau et al., 2001) originate locally from the amygdala, either from intra- or inter-nuclear sources as well as from cortical areas (Fremeau et al., 2001; Poulin et al., 2008; Andrasi et al., 2017). Numerous cortical regions supply the BLA with excitatory axon terminals, including the prefrontal, insular, higher-order sensory cortices, and ventral hippocampus, all these areas also receive reciprocal projections from the amygdala (Swanson and Petrovich, 1998; Sah et al., 2003). VGluT2-expressing boutons, a vesicular glutamate transporter type, which characterizes subcortical inputs (Fremeau et al., 2001), derive predominantly from various thalamic nuclei (Turner and Herkenham, 1991). Specifically, the LA receives thalamic inputs from the parvocellular part of the ventral posteromedial (VPMpc), the suprageniculate (SG), the medial part of the medial geniculate (MGNm), and posterior intralaminar (PIL) nuclei. In contrast, the BA collects thalamic afferents from the paraventricular (PVT), centromedial (CM), intralaminar (ILM), xiphoid (Xi) and anteriomedial (AM) nuclei. Strictly speaking, the BLA does not receive low-processed sensory inputs, as neither primary thalamic nuclei, nor primary sensory cortices project to this region. In contrast, multisensory and highly processed information is transmitted to the BLA *via* afferents from higher-order thalamic nuclei and secondary or higher-order sensory cortices besides the associative cortices (Sah et al., 2003). These anatomical constraints place the BLA circuits into the position to compare and contrast highly processed information obtained in the past with actual needs, as other cortical areas do (Alexander and Brown, 2018; Keller and Mrsic-Flogel, 2018).

At the output sites, both the LA and BA innervate a substantial part of the basal ganglia, including the nucleus accumbens, olfactory tuberculum, and posterior striatum (McDonald, 1991). Moreover, the LA and BA target the amygdalostriatal transition area (astria) and the dorsomedial striatum, respectively (McDonald, 1992; Barsy et al., 2020). Besides the basal ganglia, the BLA also projects to other striatal structures that are considered to be parts of the amygdala or rather the extended amygdala, namely the central nucleus of amygdala, interstitial nucleus of the posterior limb of the anterior commissure (IPAC), and the bed nucleus of stria terminalis (BNST; Sah et al., 2003).

In summary, distinct thalamic nuclei target the LA and BA, while both nuclei project mainly to overlapping areas of the basal ganglia (with some exceptions). At present, it is not clear if neurons in the LA and BA innervate coinciding or separate circuits within striatal structures. Future studies using advanced techniques should address this important question.

## Overview of GABAergic Cell Types in The BLA

**Figure 1:**
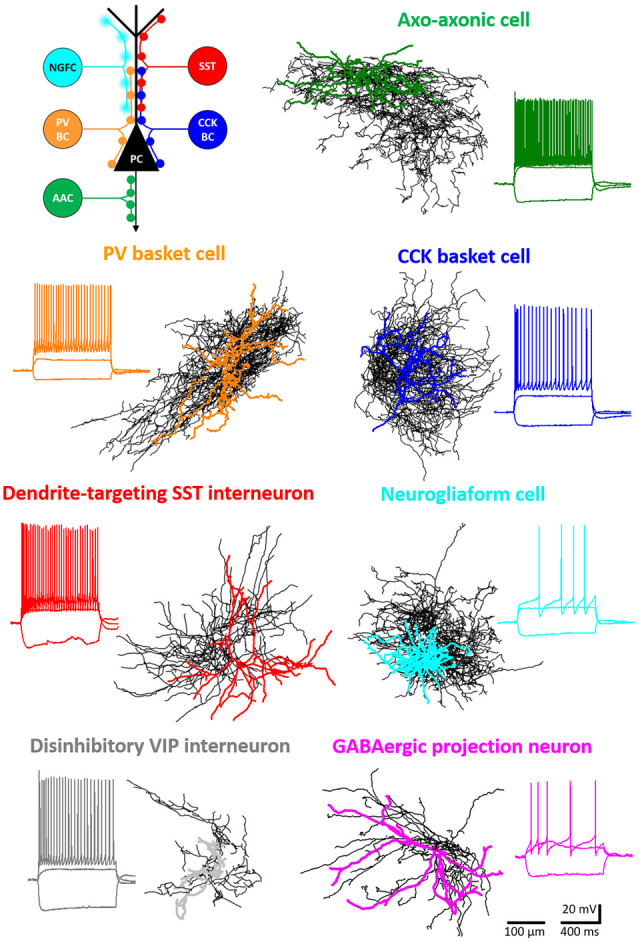
Major inhibitory cell types in the basolateral amygdala (BLA). Intracellularly labeled GABAergic cells were sampled in slice preparations and reconstructed (dendrites in color, axons in black). Voltage responses to depolarizing and hyperpolarizing step current injections are shown for each example cell. Schematic (upper left panel) shows the different membrane domains of principal cells (PC) innervated by distinct interneuron types. NGFC, neurogliaform cells expressing NPY; SST, dendrite-innervating interneurons expressing somatostatin; PVBC, parvalbumin-containing basket cells; CCKBC, cholecystokinin-expressing basket cells; AAC, axo-axonic cells.

Similar to the hippocampus and neocortex, GABAergic cells in the BLA give rise to about 20% of the total neuronal population. Specifically, in the LA 16% of all neurons are inhibitory neurons, whereas in the BA this ratio is significantly higher, 22%, as it has been recently determined (Vereczki et al., 2021). These GABAergic neurons may be categorized into four major functional groups defined by their axonal targets: (1) perisomatic region-targeting inhibitory cells innervate the soma, proximal dendrites, or axon initial segment (AIS) of principal cells and provide the most effective control of spiking activity; (2) dendrite-targeting inhibitory cells innervate primarily the dendrites of principal cells, where they can regulate dendritic computation; (3) interneuron-selective interneurons (ISI) form synaptic contacts specifically with other GABAergic cells, and thus interneurons in this category are in a position to disinhibit principal cells temporarily, permitting their activity to increase in an input-specific manner; and (4) GABAergic projection cells giving rise to both local axonal collaterals and long-range projections can control information flow between the BLA and remote regions they project to. In the listed four major GABAergic cell groups, all cardinal inhibitory cell types of cortical microcircuits have been identified (Fishell and Kepecs, 2020), which will be the subject of the present review (Figures 1, 3). Of note, this review focuses primarily on the microcircuit organization of the BLA, including single-cell features and the connectivity characteristics of individual inhibitory cell types. In addition, I cover the role GABAergic neurons play in circuit operation only in brief by providing updates as this topic has been summarized earlier in detail (Ehrlich et al., 2009; Krabbe et al., 2018). Moreover, it has to be emphasized that the majority of functional studies that aim at elucidating the role of distinct interneuron types in the amygdala use Pavlovian fear conditioning paradigm, as a model of associative learning (Fanselow and LeDoux, 1999; Maren, 2001). In this model, a conditioned stimulus (CS, often a tone) is paired several times with an unconditioned stimulus (US, a mild foot shock) during the conditioning phase. On the subsequent day, CS is presented to test the fear memory formation by monitoring the behavioral response, which is freezing in rodents. If animals are subjected to the CS several times alone, then the CS will not predict the threat anymore, i.e., the fear memory will become extinct. This extinction is likely a combination of new learning of safety and updating the original fear memory (Khalaf et al., 2018; Kida, 2019). As the amygdala plays a role in several other functions on top of fear memory formation and update, upcoming work should shed light on the role of distinct inhibitory neuron types in additional amygdala-related cognitive processes that have not been examined so far.

## Perisomatic Inhibition in Cortical Regions Originates from Three Interneuron Types

**Figure 2:**
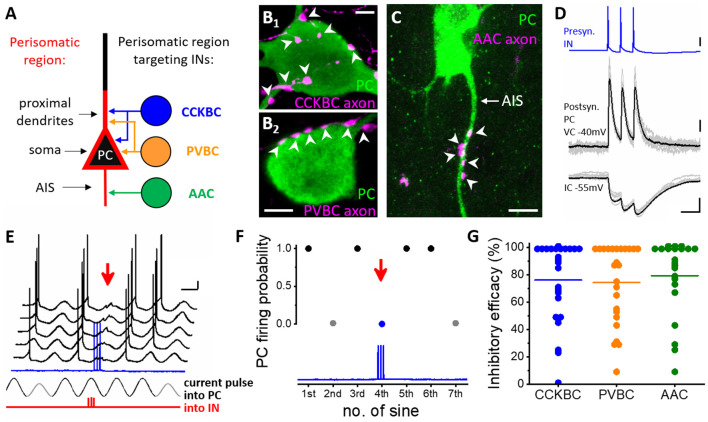
Distinct types of perisomatic region-targeting interneurons provide equally potent synaptic inhibition onto the principal cells in the BLA. **(A)** Persiomatic region is composed of the soma and the spine-free proximal dendrites as well as the axon initial segment (AIS). This region of the principal cells (PC) is innervated by three interneuron (IN) types: cholecystokinin-expressing basket cells (CCKBC), parvalbumin-containing basket cells (PVBC), and axo-axonic cells (AAC). The two basket cell types target overlapping membrane domains of principal cells, whereas their axon initial segments are selectively innervated by axo-axonic cells. **(B_1_–B_2_)** Axonal varicosities of a basket cell axon (BC, magenta, left) and **(C)** axo-axonic cell axon (AAC, magenta, right) form close appositions with the soma and proximal dendrite (arrowheads, left) and the AIS (arrowheads) of a principal cell (PC), respectively. **(D)** Three action potentials of a presynaptic interneuron (IN, in this case, a CCKBC) evoke postsynaptic responses in a principal cell (PC) recorded in voltage clamp (VC) and current clamp (IC) mode. **(E)** Testing the capacity of a perisomatic region-targeting interneuron (CCKBC) to inhibit spiking in a principal cell. Sinusoidal current trains were injected into the principal cell (PC), and three action potentials were evoked at 30 Hz in the interneuron (red, IN) 30–40 ms before the peak of the fourth cycle. Red arrow indicates the inhibitory postsynaptic potentials and lack of action potential generation in the principal cell caused by the presynaptic interneuron spike train (blue). Voltage traces are offset for clarity. **(F)** Summary plot of the experiment shown in **(D)**. Firing of the PC was prevented when the presynaptic CCKBC spiked three action potentials (red arrow). **(G)** Comparison of the inhibitory efficacy of three interneuron types targeting the perisomatic region. The inhibitory efficacy shows the probability of the suppression of spike generation in the principal cell by interneuron firing. At the population level, there is no difference in the efficacy of synaptic inhibition elicited by three perisomatic region-targeting interneuron types. Each dot represents the inhibitory efficacy obtained in a paired recording, line indicates median. This plot combines data published in (Veres et al., 2014; 2017). Scale bars: **(B_1_–B_2_**,**C)**: 5 μm; **(D)**: upper panel: 20 mV, middle panel: 50 pA, lower panel: 2 mV and 50 ms; **(E)**: 10 mV and 200 ms.

Perisomatic inhibition refers to synaptic inputs formed by GABAergic axon terminals targeting the spine-free proximal dendrites, soma, or AIS of postsynaptic principal neurons (Figure 2A; Freund and Katona, 2007; Vereczki et al., 2016). In the BA, the functional border of the perisomatic region along the individual dendrites of principal neurons can be labeled with immunostaining against the voltage-gated K^+^ channel subunit Kv2.1 (or KCNB1), which visualizes 30 μm-long proximal segments of the dendrites on average. The end of the Kv2.1-immunostained dendritic segments correspond to the steepest increase in spine density along the dendrites, which defines the extent of the perisomatic region (Vereczki et al., 2016). Remarkably, the membrane surfaces forming the perisomatic region of cortical principal neurons are predominantly, if not solely covered by GABAergic synapses (Gulyas et al., 1999; McDonald et al., 2002; Muller et al., 2006; Vereczki et al., 2016). With some notable exceptions, perisomatic inhibition in all cortical structures originates from three distinct types of GABAergic interneurons. Namely, axo-axonic cells (or chandelier cells as often called in the neocortex) form synaptic contacts specifically with the AIS of cortical principal, but not GABAergic cells (Figures 2A,C; Somogyi, 1977; Somogyi et al., 1985). Basket cells expressing either parvalbumin (PV) or cholecystokinin, and CB1 cannabinoid receptors (CCK/CB1) give rise to the vast majority of inhibitory synapses contacting the soma and the proximal dendrites (Figures 2A,B; Freund and Katona, 2007). While cortical principal cells lacking innervation from axo-axonic cells have not been reported, there are some special cases where either PV or CCK/CB1 basket cells are the only sources of GABAergic innervation on the somata and proximal dendrites of excitatory neurons (Bodor et al., 2005; Varga et al., 2010). It is worth mentioning that perisomatic inhibition in non-cortical regions, as in the cerebellum and striatum originates predominantly, if not exclusively from PV basket cells (Ito, 2006; Burke et al., 2017). As axo-axonic cells and CCK/CB1 basket cells have so far been described only in cortical areas, these perisomatic inhibitory cells are likely to be involved in neural processes specific to the cortical operation.

### Axo-Axonic Cells

This interneuron type was recognized first in the neocortex by János Szentágothai (Szentágothai and Arbib, 1974), while Péter Somogyi identified their targets as axon initial segments (Somogyi, 1977). In the BLA, the presence of these interneurons has been predicted by showing that the axon initial segments are densely covered with axon terminals forming symmetric synapses (McDonald et al., 2002), a typical feature for GABAergic synaptic junctions in cortical structures. Marco Capogna’s group was the first to demonstrate the existence of axo-axonic cells in the rat BLA (Bienvenu et al., 2012), a finding that was followed by the identification of this cell type in the mouse (Figures 2A,C; Veres et al., 2014) as well as in the monkey BLA (McDonald and Augustine, 2020). In a recent study, we have estimated that 0.8% and 1.3% of all neurons in the mouse LA and BA belong to axo-axonic cells, respectively (Vereczki et al., 2021). The majority of them (~70%) express PV, while a small fraction lacks this Ca^2+^ binding protein (Vereczki et al., 2021). Uniformly, axo-axonic cells do not express calbindin (Calb), another Ca^2+^ binding protein (Bienvenu et al., 2012; Vereczki et al., 2016; Andrasi et al., 2017; Rovira-Esteban et al., 2019), which is typically present in PV basket cells (Vereczki et al., 2006; Bienvenu et al., 2012; Andrasi et al., 2017; Rovira-Esteban et al., 2019). Thus, Calb content can be used to distinguish axo-axonic cells from PV basket cells even in the absence of axonal labeling at least in the rodent amygdala circuits.

Axo-axonic cells in the BLA have the shortest dendritic and axonal arborization in comparison to the other two types of perisomatic inhibitory cells (Vereczki et al., 2016). The axon collaterals of axo-axonic cells often display tightly packed varicosities, forming so-called cartridges, which are separated by a longer bouton-free axonal segment. As the directionality of the axon initial segments of amygdalar principal neurons seems to be random, the cartridges of axo-axonic cells do not display a “chandelier” like appearance, which is typical for these interneurons in the neocortex. Axo-axonic cells in the BA innervate their postsynaptic partners with eight-nine boutons on average, ranging between 2 and 16. The axon initial segment of principal neurons in the BA is covered by approximately 50 GABAergic terminals, therefore one may estimate that on an average five-six axo-axonic cells converge on a single principal neuron (Veres et al., 2014). On the other hand, single axo-axonic cells may innervate 600–650 principal neurons in their vicinity. This number roughly corresponds to 18–20% of all principal neurons that are present in the axon cloud of a single axo-axonic cells (Vereczki et al., 2016).

Action potentials evoked in axo-axonic cells trigger GABA_A_ receptor-mediated synaptic responses in their postsynaptic partners (Veres et al., 2014). Perforated patch recordings that only minimally alter the intracellular Cl^−^ concentrations in the recorded neurons have shown that GABA released from the axon terminals of axo-axonic cells causes hyperpolarization in postsynaptic neurons, indicating that these interneurons function as inhibitory cells in the BA (Veres et al., 2014). In line with this finding, it has been estimated that when the membrane potential of postsynaptic principal neurons was adjusted near their firing threshold, GABA release from at least 10–12 axo-axonic cell synapses was required to veto the spiking in the postsynaptic neuron. In addition, axo-axonic cell output could postpone the principal neuron spiking, if the synaptic inhibition arrived 50–150 ms prior to the would-be action potential initiation in the principal neuron. Moreover, axo-axonic cells could suppress excitatory input-driven firing, too (Veres et al., 2014). Taking into account that a single axo-axonic cells give rise to eight-nine contacts onto a given axon initial segment on an average, co-activation of 2–3 axo-axonic cells is likely sufficient to effectively control the spiking of their postsynaptic partners in the BA (Veres et al., 2014).

The analysis of the distribution of GABAergic axon terminals along the axon initial segments in the BA has uncovered that the largest density of boutons irrespective of their PV content peaked at 20–40 μm measured from the soma (Veres et al., 2014; Vereczki et al., 2021). So what is special in that region of the axon initial segment covered by GABAergic terminals with the highest density? Dual electrophysiological recordings have showed that the highest probability for action potential generation in amygdalar principal neurons overlapped with this portion of the axon initial segment. In line with this observation, the density of the immunolabeling for voltage-gated Na^+^ channel type 1.6 (Nav1.6) also peaked at 20–40 μm from the beginning of the axon initial segments. Remarkably, axo-axonic cells innervated this region with the highest likelihood, irrespective of the number of boutons given by single axo-axonic cells. These results collectively suggest that axo-axonic cells strategically position their GABAergic output synapses onto that portion of the axon initial segment, where the action potential generation has the highest probability. Although the length of axon initial segments is shorter in the LA than in the BA, the relative distance for spike generation site along the axon initial segment (densely covered by GABAergic boutons) in the LA is comparable to that observed in the BA (Vereczki et al., 2021). In summary, axo-axonic cells are in a position to efficiently control the spiking of their postsynaptic partners, as has been demonstrated in paired recordings (Veres et al., 2014).

Whole-cell recordings obtained in slice preparations showed that axo-axonic cells display a fast-spiking phenotype, have low input resistance, and fast membrane time constant (Barsy et al., 2017). *In vivo* electrophysiological recordings showed that these interneurons have narrow spikes recorded extracellularly, a feature based on which one cannot distinguish axo-axonic cells from PV basket cells (or from additional GABAegic cell types; Bienvenu et al., 2012). Axo-axonic cells in the BLA often fire a burst of action potentials at 300 Hz or even at higher rates, a characteristic feature, which distinguishes them from PV basket cells (Barsy et al., 2017). As the maximal firing rate for PV basket cells has been reported to be less than 200 Hz (Woodruff and Sah, 2007; Barsy et al., 2017), the difference in the burst frequency between axo-axonic cells and PV basket cells may be used as a “biomarker” to differentiate between these two narrow spiker interneurons *in vivo* recordings.

Our knowledge is very limited regarding the *in vivo* firing features of axo-axonic cells in the BLA. In a study obtained in anesthetized rats, noxious stimuli like a tail pinch or electrical shock robustly elevated the firing of all axo-axonic cells tested (Bienvenu et al., 2012), similarly to that observed in the medial prefrontal cortex (Massi et al., 2012). These results may suggest that these GABAergic cells can play a role in controlling aversive emotional states evoked by painful stimulation. In another recent study, the role of amygdalar axo-axonic cells in emotional memory formation has been examined by disrupting GABAergic synaptic contacts along the axon initial segments, while sparing those that contacted the soma. This selective manipulation has been achieved by knocking down the cell adhesion molecule neurofascin, which stabilizes axo-axonic GABAergic synapses along the axon initial segment (Kriebel et al., 2011). The results of the neurofascin knockdown have shown that the fear extinction, but not the cued fear conditioning, was impaired upon reducing the number of GABAergic synapses contacting the axon initial segments (Saha et al., 2017). Future research using selective modulation of axo-axonic cell activity may elucidate more precisely the role of these GABAergic interneurons in amygdala operation.

### PV Basket Cells

Basket cells in cortical structures were described by Santiago Ramón y Cajal using Golgi impregnation technique (Ramón y Cajal, 1899). The presence of PV in basket cell axon terminals was demonstrated first in the hippocampus (Kawaguchi et al., 1987) and neocortex (Hendry et al., 1989) using immunocytochemistry. In subsequent studies, electron microscopic investigations revealed that in the BLA, axon terminals expressing PV contained GABA and formed synaptic contacts with somata and proximal dendrites of principal cells (Figures 2A,B; Sorvari et al., 1996; Smith et al., 1998), distinguishing features of PV basket cells. These interneurons comprise about 2.2% and 4.7% of all neurons in the mouse LA and BA, respectively (Vereczki et al., 2021). At the population level, around 50% of PV basket cell output synapses contact the soma and proximal dendrites, i.e., the perisomatic region. The other half of the axonal varicosities of this basket cell type targets distal dendrites, showing an exponential decrease in the contact number toward the tip of dendrites (Smith et al., 1998; Muller et al., 2005; Vereczki et al., 2016; Veres et al., 2017). Although there is a large variance in the ratio of perisomatic vs. dendritic targets of individual PV basket cells, no dendrite-targeting PV interneurons, like PV-containing bistratified cells in the CA1 region of the hippocampus (Halasy et al., 1996) have been identified in the BLA, in spite of the fact that some PV interneurons express NPY (Vereczki et al., 2021), a characteristic marker for CA1 bistratified cells (Klausberger et al., 2004). The largest fraction of perisomatic GABAergic inputs (~40%) received by amygdalar principal neurons originate from PV basket cells, a ratio that corresponds to 90–95 boutons (Vereczki et al., 2016). As single PV basket cells give rise to four-five contacts on the perisomatic region of principal neurons on average, 18–24 PV basket cells should converge on the soma and proximal dendrites of single principal neurons (Vereczki et al., 2016; Veres et al., 2017). On the other hand, a single PV basket cell may innervate 950–1,000 principal neurons, corresponding to roughly 10% of all principal neurons within the area of their axonal arbor. These refined estimates are based on the combined data published recently (Vereczki et al., 2016, 2021; Veres et al., 2017).

PV basket cells have variable soma size, but uniformly give rise to a multipolar dendritic tree (McDonald and Betette, 2001; Mascagni et al., 2009; Vereczki et al., 2016). Their membrane surface is densely covered by synaptic inputs (Smith et al., 1998). The excitatory inputs they receive on their somata and dendrites (McDonald et al., 2005; Andrasi et al., 2017) are more numerous than inhibitory contacts (Smith et al., 1998; in the hippocampus it has been estimated that more than 15,000 excitatory and 3,000 inhibitory synapses contact single PV interneurons; Gulyas et al., 1999). Excitatory synaptic inputs onto PV basket cells are mediated primarily *via* Ca^2+^-permeable AMPA receptors, the number of which can be increased upon tetanic stimulation causing long-term potentiation (Mahanty and Sah, 1998). These data suggest that the efficacy of excitatory inputs received by PV basket cells in the amygdala can be changed depending on the activity level linked to distinct environmental challenges. Notably, the induction features of long-term potentiation at excitatory synapses studied in fast spiking PV interneurons (the majority of which were likely basket cells) may be different in the LA and BA (Lucas et al., 2016; Polepalli et al., 2020).

Recordings obtained in perforated patch configuration have revealed that PV basket cells are inhibitory cells, as the reversal potential of their GABA_A_ receptor-mediated postsynaptic responses monitored in amygdalar principal neurons was more hyperpolarized than the resting membrane potential of the postsynaptic neurons (Veres et al., 2017). Interestingly, the reversal potential of postsynaptic potentials from basket cells has more negative values than those originated from axo-axonic cells (Veres et al., 2014). This difference in reversal potentials is in accord with results obtained in hippocampal pyramidal cells using GABA uncaging (Khirug et al., 2008). The reason for the difference in the reversal potentials may be explained, at least in part, by the fact that in the plasma membrane of axon initial segments the neuron-specific potassium-chloride cotransporter 2 (KCC2), a major controller for intracellular Cl^−^ concentrations (Kaila et al., 2014) is far less abundant than along the soma and dendrites (Baldi et al., 2010).

Paired recordings in acute slice preparations uncovered that 8–12 perisomatic contacts of basket cells are needed to block the spiking in amygdalar principal neurons when their membrane potential is adjusted near the firing threshold. Furthermore, the timing of the firing can be postponed within a 110 ms-long interval by PV basket cells (Veres et al., 2017). These observations indicate that simultaneous activation of two-three PV basket cells is necessary to suppress the postsynaptic spiking. It is important to note that the control of spike generation by basket cells is achieved primarily *via* their perisomatic inputs, while their inputs onto the distal dendrites may have only limited contribution to this effect (Veres et al., 2017). Although, the function of dendritic inhibition provided by basket cells has not been investigated yet, they may play a role in influencing local signaling (Miles et al., 1996; Mullner et al., 2015).

PV basket cells receive innervation both from intra- and extra-amygdalar sources. Intra-amygdalar sources include principal cells and interneurons as well (Smith et al., 2000; McDonald et al., 2005; Muller et al., 2005; Andrasi et al., 2017), with inhibitory input originating from other PV basket cells (Muller et al., 2005; Woodruff and Sah, 2007; Andrasi et al., 2017; Krabbe et al., 2019), and SST and VIP interneurons (Krabbe et al., 2019). Extra-amygdalar input is provided by the frontal, auditory, rhinal and insular cortices, ventral hippocampus, different thalamic nuclei, basal forebrain, dorsal raphe, locus coeruleus, and dopaminergic neurons, uncovered by the use of monosynaptic rabies tracing (Lucas et al., 2016; Krabbe et al., 2019), electron microscopy (Smith et al., 2000; Muller et al., 2007b, 2011; Pinard et al., 2008; McDonald et al., 2011), or optogenetics (Polepalli et al., 2020).

Similarly to axo-axonic cells, PV basket cells show a fast-spiking phenotype, although a substantial variability can be acknowledged in their voltage responses upon depolarizing current step injections (Rainnie et al., 2006; Woodruff and Sah, 2007; Barsy et al., 2017; Polepalli et al., 2020). These interneurons typically have low input resistance and fast membrane time constant, and display a high firing rate in the intact brain (Woodruff and Sah, 2007; Bienvenu et al., 2012; Wolff et al., 2014; Barsy et al., 2017). In contrast to axo-axonic cells, however, PV basket cells show variable responses to noxious stimulations, as some are excited, while others are inhibited or non-responsive in anesthetized rats (Bienvenu et al., 2012). Comparable diverse responses in PV interneuron populations upon both CS and US stimuli during fear conditioning were observed in behaving mice using tracing Ca^2+^ transients (Krabbe et al., 2019). The vast majority of PV interneurons responded to the US: 80% of them were found to elevate their activity, whereas 20% reduced it. Fewer PV interneurons responded to the US-associated CS+ (~75%) and US-independent CS− (~50%) presentations. Of the CS-responsive PV interneurons around 2/3 elevated their activity, the others decreased it (Krabbe et al., 2019). Considering that the majority of PV interneurons are basket cells, these results may imply that distinct populations of PV basket cells may belong to various amygdalar sub-circuits acting distinctly, but in concert to fulfill the optimal computation during behavioral challenges. In a different study, optogenetic manipulation of PV interneuron function during associative fear learning revealed that time-locked inhibition of the PV interneuron population during CS and US stimuli caused improvement or impairment of fear memory formation, respectively (Wolff et al., 2014). Using chemogenetics, it has been shown that PV interneurons may control the number of neurons that participate in memory formation (Morrison et al., 2016), further supporting the role of these interneurons in associative learning. In addition, PV basket cells may also control extinction learning, as “fear” neurons in the amygdala that are activated by associative fear memory formation but are inhibited during extinction training (Herry et al., 2008) received increased innervation from these perisomatic inhibitory cells upon repetitive presentation of CS that no longer signed threat (Trouche et al., 2013).

In summary, PV basket cells are key circuit elements in the BLA complex, where they contribute to fear memory processes. Subsequent studies will likely uncover in the close future how these inhibitory interneurons control other amygdala-linked neural operations.

### CCK/CB1 Basket Cells

In addition to GABAergic axon terminals containing PV, other boutons that express CCK were found to form synaptic contacts with the perisomatic region of cortical principal cells (Hendry et al., 1983; Nunzi et al., 1985), demonstrating that two distinct basket cell types participate in perisomatic innervation. CCK basket cells have a unique feature, as their axonal varicosities are decorated with CB1 cannabinoid receptors shown first by István Katona and his colleagues (Katona et al., 1999b). In the BLA, CCK/CB1 basket cells are also present both in the rodent (Katona et al., 2001; McDonald and Mascagni, 2001; Vereczki et al., 2016; Rovira-Esteban et al., 2017) and in the monkey (McDonald, 2021), giving rise to 0.9% of all neurons in the LA and 2.1% in the BA in the mouse BLA (Vereczki et al., 2021). CCK/CB1 basket cells typically have large somata and multipolar dendritic trees both in rodents (Mascagni and McDonald, 2003; Vereczki et al., 2016; Rovira-Esteban et al., 2017) and monkey (McDonald and Mascagni, 2019). The neurochemical content of CCK/CB1 basket cells is variable, as they may express vesicular glutamate transporter type 3 (VGluT3) and Calb in largely non-overlapping subpopulations (Omiya et al., 2015; Rovira-Esteban et al., 2017). In some of these basket cells, VIP may also be expressed (Omiya et al., 2015; Rhomberg et al., 2018). Of note, a recent study has revealed that the Ca^2+^ binding proteins NECAB1 and 2 are expressed in CCK/CB1, but not PV basket cells in cortical structures, including the BLA (Miczan et al., 2021). At the population level, half of the output synapses of CCK/CB1 basket cells target the perisomatic region (similarly to PV basket cells), whereas the other half contacts distal dendrites with a progressive decline in density toward the tip of dendrites (Figures 2A,B; Veres et al., 2017). One-third of perisomatic GABAergic inputs onto amygdalar principal neurons originates from CCK/CB1 basket cells, a ratio that corresponds to 65–70 boutons (Vereczki et al., 2016). As single CCK/CB1 basket cells contact the perisomatic region of principal neurons *via* three-four contacts on average, 16–23 CCK/CB1 basket cells should innervate single principal neurons perisomatically (Vereczki et al., 2016; Veres et al., 2017). On the other hand, individual CCK/CB1 basket cells may target 680–730 principal neurons, corresponding to approx. 10% of all principal neurons within the volume of their axonal arbor (Vereczki et al., 2006, 2021; Veres et al., 2017).

CCK/CB1 basket cells receive both excitatory and inhibitory synapses on their membrane surface with a substantially lower density in comparison to PV basket cells (Matyas et al., 2004; Andrasi et al., 2017). Our knowledge regarding the sources of amygdalar CCK/CB1 basket cell inputs is limited. It has been shown that they receive excitatory innervation from amygdalar principal cells, whereas their synaptic inhibition arrives from other CCK/CB1 basket cells and VIP-expressing ISI (Andrasi et al., 2017; Rhomberg et al., 2018). Previous studies obtained in the hippocampus have observed that excitatory synaptic inputs of CCK/CB1 basket cells show no long-term changes using induction protocols in acute slices that readily induced long-term potentiation or long-term depression at glutamatergic afferents recorded from PV basket and axo-axonic cells (Nissen et al., 2010; Szabo et al., 2012). However, environmental challenges may alter excitatory inputs on CCK/CB1 basket cells on a longer time scale, a hypothesis that needs to be tested.

Postsynaptic responses of CCK/CB1 basket cells are mediated *via* GABA_A_ receptors (Vogel et al., 2016), although their high spiking rates may also cause the activation of GABA_B_ receptors on the postsynaptic targets (Booker et al., 2013). As revealed by perforated patch recordings, CCK/CB1 basket cell-evoked postsynaptic events in amygdalar principal neurons are inhibitory, similarly to the two other perisomatic inhibitory cell types (Veres et al., 2017). Estimated from whole-cell paired recordings, 8–12 axon terminals of CCK/CB1 basket cells are needed to prevent spiking of amygdalar principal neurons, with membrane potentials set near the firing threshold (Figures 2D–F). Similarly to PV basket cells, CCK/CB1 basket cells are also able to postpone the spiking of principal neurons by 110 ms, on average. As individual CCK/CB1 basket cells were assessed to give three-four perisomatic contacts to principal neurons on average, simultaneous discharge of two-four basket cells of this type is necessary to control the spiking of their postsynaptic partners effectively (Veres et al., 2017).

In contrast to the two other perisomatic inhibitory interneurons, the firing of CCK/CB1 basket cells show accommodation, at least at young ages (Jasnow et al., 2009; Barsy et al., 2017), a spiking phenotype that develops to more clustered action potential firing with age (Rovira-Esteban et al., 2019). Typically, these basket cells have higher input resistance, slower membrane time constant, and often display a sag in voltage responses evoked by hyperpolarizing current steps, indicative of the expression of h-current (Jasnow et al., 2009; Vogel et al., 2016; Rovira-Esteban et al., 2017).

The functional role of CCK/CB1 basket cells in neural operation is unclear. The primary reason for this lack of knowledge lies in the fact that currently there is no available tool to manipulate the function of these basket cells without affecting other inhibitory neuron types even by using intersectional viral strategy (Rovira-Esteban et al., 2019). Recent studies, however, provide some hints for the role of CCK/CB1 basket cells in the control of fear memory. For instance, these basket cells can differentially regulate the activity of amygdalar principal cells that project to distinct parts of the prefrontal cortex. In paired recordings, it has been revealed that CCK/CB1 basket cells evoke comparable unitary inhibitory postsynaptic events in principal neurons irrespective of whether they project to the prelimbic or infralimbic cortex. However, there was a striking difference in the CB1 receptor-mediated inhibition of GABA release from the axon terminals of these basket cells upon the activation of postsynaptic neurons with distinct projections (Vogel et al., 2016). Earlier it has been discovered that endocannabinoids, endogenous ligands of CB1 receptors, are released from the postsynaptic neurons in an activity- dependent manner, causing suppression of neurotransmitter release from presynaptic varicosities (Wilson and Nicoll, 2001). In the BA, those principal neurons that project to the prelimbic cortex could liberate themselves fully from inhibition provided by CCK/CB1 basket cells in an activity- and CB1 receptor-dependent manner, whereas those principal neurons that innervate the infralimbic cortex could reduce this source of synaptic inhibition only partially upon depolarization (Vogel et al., 2016). Thus, CCK/CB1 basket cells can contribute to the dynamic control of the BA output. In addition, other indirect evidence (like correlative changes in bouton numbers and behavior) suggests that these basket cells may critically contribute to extinction learning (Ruehle et al., 2013; Trouche et al., 2013; Rovira-Esteban et al., 2019) and the control of synaptic plasticity demonstrated in slice preparations (Azad et al., 2008). After developing appropriate tools in the future, our knowledge should be substantially advanced regarding the CCK/CB1 basket cell function in amygdala operation.

## Microcircuit Organization by Perisomatic Inhibitory Cells and Principal Neurons

By comparing the efficacy of inhibition, i.e., the ability of a principal neuron to fire if a monosynaptically connected perisomatic inhibitory cell discharges action potentials under a given condition, recent investigations have revealed that all three types of perisomatic inhibitory cells can control the spiking of postsynaptic principal neurons in the amygdala microcircuits to a similar extent (Figure 2G; Veres et al., 2014, 2017; Andrasi et al., 2017). This surprising observation is in line with the fact that the magnitude of unitary events originating from distinct types of perisomatic inhibitory neurons does not differ substantially in the BA. In contrast, the unitary events CA3 pyramidal cells receive from axo-axonic cells have significantly larger peak amplitude than those arriving from basket cells in the hippocampus (Szabo et al., 2010). In the prefrontal cortex, the peak amplitude of the unitary events between PV basket cells and pyramidal cells was found to be significantly larger in comparison to those recorded in pairs of axo-axonic cells and pyramidal cells or CCK/CB1 basket cells and pyramidal cells (Fekete et al., 2019). These regional differences in the magnitude of unitary inhibitory postsynaptic currents may indicate a more equalized impact for the three perisomatic inhibitory cell types on neural activity in some cortical regions like the BA, while in other regions, one perisomatic inhibitory cell type may have a more profound effect on microcircuit operation at the single-cell level. Clearly, more work is needed to understand the logic and significance of the differences in the efficacy of inhibition provided by the distinct perisomatic inhibitor cell types on neuronal functions.

Consequently, if the synaptic inhibition received by the amygdalar principal neurons from the three perisomatic inhibitory cell types is similar, then the synaptic inputs of these GABAergic cells should differ, otherwise, they cannot fulfill distinct functions in circuit operation as predicted earlier (Freund and Katona, 2007). Indeed, a substantially smaller population of principal neurons can discharge PV basket cells than CCK/CB1 basket cells (Andrasi et al., 2017). Several factors underlie this differential excitation, including the dissimilarity in the peak amplitude of unitary excitatory events and the number of excitatory inputs received by PV basket cells and CCK/CB1 basket cells. Distinct excitation of PV basket cells and CCK/CB1 basket cells may dictate the differential recruitment of these GABAergic cell types during various functions, a necessary prerequisite to fulfill their dedicated roles in amygdala networks.

Another surprising observation is related to the connectivity among these three perisomatic inhibitory cell types. With two independent methods, using paired recordings and immunocytochemistry, it has been uncovered that PV basket cells innervate each other as well as axo-axonic cells with high probability, but avoid CCK/CB1 basket cells. On the other hand, CCK/CB1 basket cells also target each other with a high probability as well as axo-axonic cells, but do not innervate PV basket cells. Axo-axonic cells do not innervate each other or any basket cells (Andrasi et al., 2017). Yet, axo-axonic cells are readily coupled *via* gap junctions, forming a syncytium (Andrasi et al., 2017). Similarly, paired recordings and electron microscopy showed that there is a high probability of finding a gap junction coupling among PV basket cells (Muller et al., 2005; Woodruff and Sah, 2007; Andrasi et al., 2017) or among CCK/CB1 basket cells, but the two basket cell types do not communicate *via* electrical synapses either (Andrasi et al., 2017). Thus, in the amygdala, two independent basket cell networks operate in parallel, which are activated distinctly *via* principal neurons. Notably, this wiring principle of perisomatic inhibitory cells and principal neurons is not unique for the amygdala, as a similar connectivity matrix appears to exist in the CA3 region of the hippocampus (Kohus et al., 2016). At present, it is unknown why this type of wiring diagram is formed at least in two cortical structures, but it may be indicative of a functional dichotomy between the two basket cell types. For instance, PV basket cells may serve as a clock in cortical networks (Freund and Katona, 2007), as together with principal neurons they can generate highly precise oscillatory activities like gamma oscillations without the contribution of axo-axonic cells or CCK/CB1 basket cells (Gulyas et al., 2010). In contrast, CCK/CB1 basket cells can control the spiking of principal neurons by providing profound inhibition on principal neurons that show low activity, while allowing the spiking of those that can release endocannabinoids upon high firing (Zhu and Lovinger, 2005; Vogel et al., 2016), and therefore likely carry important information. Thus, PV basket cells control “when” to spike, while CCK/CB1 basket cells regulate “who” can fire. The control of principal cell spiking with these two distinct purposes can be achieved with independent networks of basket cells most efficiently.

## Dendritic Inhibition

The term dendritic inhibition refers to GABAergic inputs forming synaptic contacts with dendrites (Miles et al., 1996). In cortical regions, two distinct types of GABAergic interneurons target preferentially the dendritic tree of principal neurons. SST (or SOM) interneurons innervate the distal dendrites of pyramidal cells (Katona et al., 1999a; Wang et al., 2004), and are considered to control dendritic information processing in a feedback manner (Miles et al., 1996; Murayama et al., 2009), as these GABAergic cells are primarily excited by the local axon collaterals of principal neurons (Blasco-Ibanez and Freund, 1995; Maccaferri and McBain, 1995). Carlo Martinotti was the first to report an interneuron that had a massive ascending axonal projection reaching even layer 1 (Scarani et al., 1996), a morphology that was clearly distinct from that of basket cells. In the hippocampus, Chris McBain and his colleagues described first an interneuron, the so-called OLM-cell that had an ascending axonal arbor far away from its soma and dendrites (McBain et al., 1994). Both Martinotti cells and OLM cells were found to express SST (Kawaguchi and Kubota, 1996; Katona et al., 1999a). SST interneurons provide GABA_A_ receptor-mediated postsynaptic responses onto their target neurons (Maccaferri et al., 2000). In contrast, the other source of dendritic inhibition originating from neurogliaform cells, reported first by Ramon y Cajal (Ramón y Cajal, 1899), supplies the microcircuits typically with slow and long-lasting inhibition, composed of both GABA_A_ and GABA_B_ receptor-mediated postsynaptic inputs (Tamas et al., 2003; Price et al., 2008). Both types of dendrite-targeting interneurons are present in the BLA in a similar quantity (McDonald, 1985; Amaral et al., 1989; McDonald et al., 1995; McDonald and Mascagni, 2002; Manko et al., 2012). SST interneurons form 1.3% of all neurons in the LA and 4% of all neurons in the BA, whereas neurogliaform cells make up 1.8% and 3.5% of the total neuronal population in the LA and BA, respectively (Vereczki et al., 2021).

### Dendrite-Targeting SST Inhibitory Cells

SST interneurons have smooth or sparsely spiny dendrites, give rise to dense local axonal arborization (Vereczki et al., 2021) and often express Calb and/or neuropeptide Y (McDonald, 1989; McDonald and Mascagni, 2002) but lack nNOS (Vereczki et al., 2021). The typical firing of SST interneurons induced by depolarizing current injection shows accommodation and a sag appears in negative voltage responses evoked by hyperpolarizing current steps, indicative of the activation of the h-current (Zemankovics et al., 2010; Unal et al., 2020; Vereczki et al., 2021). These interneurons preferentially innervate small-caliber dendrites, and to a lesser extent, spines of amygdalar principal neurons (Muller et al., 2007a; Vereczki et al., 2021), similarly to that observed in the hippocampus (Katona et al., 1999a) and neocortex (Wang et al., 2004), which explains the slow rising phase of their postsynaptic responses measured at the soma (Wolff et al., 2014; Krabbe et al., 2019; Unal et al., 2020). As amygdalar principal neurons receive their excitatory inputs on their dendrites (Smith and Pare, 1994; Brinley-Reed et al., 1995; Pare et al., 1995; Vereczki et al., 2016; Amir et al., 2019), SST interneurons are in a key position to control the efficacy of excitatory inputs and thus the plastic changes in synaptic strength and dendritic excitability. Indeed, recently it has been demonstrated that the spiking of amygdalar principal neurons evoked by synaptic excitation could be suppressed by activation of SST inhibitory cells (Wolff et al., 2014). This activation can even lead to gating long-term potentiation, at least at excitatory synapses of prefrontal-basal amygdala afferents (Ito et al., 2020).

SST interneurons receive innervation from both intra- and extra-amygdalar sources. Their excitatory inputs from local principal neurons show short-term facilitation (Unal et al., 2020), which ensures a time window for a potential discharge before the feedback inhibition reaches the dendritic tree (Unal et al., 2020), similarly to that observed in other cortical regions (Pouille and Scanziani, 2004). SST interneurons are also part of inhibitory circuits in the BLA, as they innervate and are targeted by both PV and VIP interneurons (Krabbe et al., 2019). In addition to SST interneurons displaying accommodating firing and sag in their voltage responses upon negative step current injections, many SST interneurons showing rather a fast-spiking phenotype and no sag were sampled in transgenic mice generated by crossing Sst-Cre mice with a reporter mouse line (Guthman et al., 2020; Unal et al., 2020). Interestingly, such fast-spiking interneurons were rarely found among SST interneurons if they were visualized by a viral strategy in Sst-Cre mice (2 out of 31; Vereczki et al., 2021). Importantly, such fast-spiking SST interneurons may express PV (Vereczki et al., 2021), raising the possibility that during development Sst gene may be temporarily active in a population of PV interneurons, leading to the expression of reporter proteins in them, if interneurons are labeled by crossing Sst-Cre mice with a reporter mouse line. In line with this hypothesis, fast-spiking SST interneurons were shown to mediate feedforward inhibition in the amygdala (Guthman et al., 2020), a typical trait of PV interneurons (Smith et al., 2000; Hu et al., 2014; Lucas et al., 2016). In contrast, SST interneurons with accommodating firing were readily recruited by amygdalar principal neurons in a feedback manner (Unal et al., 2020), a feature that characterizes dendrite-targeting SST interneurons in cortical structures (Blasco-Ibanez and Freund, 1995; Maccaferri and McBain, 1995; Murayama et al., 2009). Clearly, further investigations are needed to clarify the neurochemical content, postsynaptic targets, and wiring features of fast-spiking SST interneurons in the amygdala.

Using monosynaptic rabies tracing the inputs onto SST inhibitory cells has recently been examined. It has been found that these GABAergic neurons receive innervation both from cortical areas (including the auditory, insular, piriform and medial orbital cortex, and ventral hippocampus) and subcortical regions (including the basal forebrain, thalamus, and dorsal raphe; Krabbe et al., 2019). As this approach does not allow the separation of monosynaptic inputs received by SST interneurons and SST projection cells (see later), more specific investigations should be conducted in the future to uncover the long-range inputs onto SST GABAergic cell types.

A recent elegant study using imaging techniques for tracking Ca^2+^ transients has investigated the response of SST inhibitory cells in the BA during fear conditioning. The authors observed that 60–70% of these GABAergic neurons changed their Ca^2+^ signals upon presentation of US, CS+, and CS−. Interestingly, half of the responsive SST inhibitory neurons decreased, whereas the other half increased their activity irrespective of the presented stimulus (Krabbe et al., 2019). Furthermore, optogenetic interventions have revealed that SST inhibitory cells in the BA may control associative memory formation bidirectionally. Inhibition and activation of SST inhibitory cell activity accompanied by CS presentation increased and reduced, respectively, the freezing levels during fear memory retrieval. Importantly, CS-evoked principal neuron firing was enhanced by inhibiting SST inhibitory cells, whereas it was suppressed by exciting these GABAergic cells (Wolff et al., 2014). These data strongly support the view that dendrite-targeting SST interneurons effectively control the formation of associative memories at the amygdala level by altering dendritic function. Moreover, SST interneurons in the amygdala may be involved in discriminative learning as well. A very recent study has uncovered that SST inhibitory cells in the amygdala were activated specifically during learned non-threatening cues. This enhanced activity was dependent on the prelimbic cortex and promoted the discrimination of non-threat stimuli. Thus, the prefrontal cortex may control amygdala function during fear discrimination *via* engaging SST interneurons (Stujenske et al., 2021).

However, one has to keep in mind that the use of Sst-Cre mice does not allow to selectively monitor and manipulate the operation of dendrite-targeting SST interneurons exclusively. Although in Sst-Cre mice, the majority of SST GABAergic cells both in the LA and BA likely belong to this interneuron category (60% and 75%, respectively; Vereczki et al., 2021), yet the remaining SST inhibitory cells express neuronal nitric oxide synthase (nNOS) that characterizes GABAergic cells with long-range projections to extra-amygdalar areas (see below). Therefore, further studies using a more selective approach will be needed to unequivocally identify the role of dendrite-targeting SST interneurons in distinct amygdala functions.

### Neurogliaform Cells

These interneurons, similarly to that observed in the hippocampus and neocortex, have short, frequently ramifying sparsely spiny dendrites, very dense local axonal arbor, and contain neuropeptide Y (NPY; Manko et al., 2012); in addition, a few of them express CCK (Rovira-Esteban et al., 2019), nNOS at a low level (Vereczki et al., 2021), or even SST (Manko et al., 2012). Similarly to other cortical areas, these GABAergic interneurons in the amygdala generate slow postsynaptic inhibitory responses mediated *via* GABA_A_ and GABA_B_ receptors (Manko et al., 2012; Rovira-Esteban et al., 2019), making them very efficient regulators of circuit operation (Tamas et al., 2003; Olah et al., 2009; Abs et al., 2018). Structural features of how neurogliaform cells contact other neurons may explain, at least partially, the typical slow postsynaptic responses. Electron microscopic studies revealed that a significant portion of axon terminals of neurogliaform cells do not form classical tight synaptic contacts with the target elements, instead, the axonal boutons appose the neural profiles from a distance, allowing the released GABA to reach far beyond the potential postsynaptic specialization (Tamas et al., 2003; Manko et al., 2012). This spill-over of GABA for large distances in the extracellular milieu can activate GABA_B_ receptors located both postsynaptically and presynaptically, even on neighboring axon terminals (Olah et al., 2009). So far, neurogliaform cells have been identified as a main source for evoking pronounced postsynaptic GABA_B_ receptor-mediated responses in cortical structures (Tamas et al., 2003). They have been shown to be activated in a feedforward manner in the hippocampus (Price et al., 2008), prefrontal cortex (Jackson et al., 2018), or auditory cortex (Abs et al., 2018), yet there is no data available on how these GABAergic interneurons are recruited during microcircuit operation in the amygdala. Neurogliaform cells often display a late-spiking phenotype and a pronounced after-hyperpolarization following the spike that is indistinguishable from the action potential of principal neurons regarding their spike width (Manko et al., 2012; Rovira-Esteban et al., 2019; Vereczki et al., 2021). This latter spike characteristic makes it challenging, if not impossible, to separate extracellularly detected spikes of neurogliaform cells from principal neurons, and on top of it, both types of neurons fire typically with a moderate rate *in vivo* (Bienvenu et al., 2012; Manko et al., 2012). This drawback restrains us from revealing the activity of neurogliaform cells during distinct behaviors without optical tagging or *post hoc* anatomical identification following juxtacellular or intracellular recordings (Manko et al., 2012). As so far no available combination of techniques has made it possible to selectively interfere with the function of neurogliaform cells in the amygdala, their contribution to circuit operation during distinct behaviors remains unknown.

## Disinhibitory Interneurons Expressing VIP/CR

GABAergic interneurons that specifically innervate other GABAergic cells have been recognized first in the hippocampus and dentate gyrus by the group of Tamás Freund (Acsády et al., 1996; Gulyas et al., 1996; Hájos et al., 1996). Using neuroanatomical techniques, they discovered that GABAergic interneurons expressing VIP and/or CR form synaptic contacts predominantly, if not exclusively on other GABAergic cells, providing the structural basis for the presence of a disinhibitory circuitry in cortical networks. More than 15 years later, Adam Kepecs and his group presented the first functional proof for the existence of such disinhibitory networks (Pi et al., 2013). Since then, numerous studies conducted in several cortical areas have shown that VIP interneurons that lack CB1 expression are fundamental elements of cortical circuits, providing disinhibition of excitatory principal neurons universally in cortical structures (Fishell and Kepecs, 2020). Accordingly, Rhomberg et al. (2018) have shown that in the LA and BA, VIP interneurons that do not express CB1 specifically target other GABAergic interneurons. These VIP interneurons form the largest fraction among GABAergic cells in the BLA (4.8% and 6.9% of all neurons in the LA and BA, respectively; Vereczki et al., 2021), have short dendrites and their axons are confined to the close vicinity of the soma (Rhomberg et al., 2018). Using optogenetics and viral tracing in VIP-Cre mice combined with confocal microscopy, it has been revealed that VIP interneurons innervate GABAergic cells that express PV or SST in addition to CCK+ basket cells and other VIP interneurons (Rhomberg et al., 2018; Krabbe et al., 2019). Of note, neurogliaform cells are rarely among the targets of VIP interneurons (Rhomberg et al., 2018), similarly to that observed in the auditory cortex (Abs et al., 2018). At present, however, it is not clear how many VIP/CB1-expressing basket cells are labeled among all VIP interneurons in VIP-Cre mice. Based on indirect evidence, it seems safe to assume that the ratio of non-disinhibitory VIP interneurons may not be substantial among viral infected neurons in VIP-Cre mice 4–5 weeks after AAV injection, as no VIP basket cells have been recorded so far using this approach. In contrast, when VIP-Cre mice were crossed with the RCL_ChR2/EYFP mouse line Ai32, CB1 sensitive postsynaptic responses were readily recorded in amygdalar principal neurons (Rhomberg et al., 2018). These observations may imply that a longer time is needed for the virus infection of VIP/CB1 basket cells than of VIP interneuron-specific interneurons.

VIP interneurons receive innervation from distinct extra-amygdalar sources and from local principal neurons, although this has not been demonstrated directly. Using monosynaptic rabies tracing, a wide range of cortical and subcortical areas have been identified as an input region to VIP interneurons. Specifically, neurons located in the auditory, insular, and rhinal cortices, ventral hippocampus, basal forebrain, and also in different thalamic nuclei provide innervation onto VIP interneurons (Krabbe et al., 2019). In addition, local GABAergic afferents also contribute to their inputs: VIP interneurons may innervate each other and receive GABAergic inputs from PV basket cells, SST inhibitory cells, and CCK/CB1 basket cells (Rhomberg et al., 2018; Krabbe et al., 2019). At present, it is unknown whether neurogliaform cells can influence the function of VIP interneuron-selective interneurons.

An already mentioned study has provided insights into the function of VIP interneurons in the amygdala during fear learning as well (Krabbe et al., 2019). Using *in vivo* imaging of Ca^2+^ activity in freely moving mice the authors observed that at the beginning of the conditioning the majority of VIP interneurons increased their activity when the US was delivered. As the conditioning progressed, VIP interneurons responded less to the US, and instead, they began to be activated by the CS. As a proof for the disinhibitory function of VIP interneurons, the activity of principal neurons, which was normally elevated by the US, could be significantly reduced if the elevated activity of VIP interneurons induced by the CS was suppressed by optogenetics. These results clearly show that: (i) VIP interneurons have disinhibitory function in the BLA, (ii) they are strongly activated by salient stimuli and (iii) their activity changes during conditioning in a way that is characteristic for neurons signaling prediction errors. Although this study has not examined the sources of afferents onto VIP interneurons that may excite them during the US presentation, it is tempting to speculate that cholinergic cells in the basal forebrain, neurons that reliably and profoundly discharge upon US presentation (Hangya et al., 2015) can contribute to driving the spiking of VIP interneurons, as these cholinergic connections have been demonstrated experimentally (Krabbe et al., 2019). In addition, thalamic afferents may also contribute to the US-induced excitation of VIP interneurons, as both midline and posterior thalamic neurons are activated by the US (Zhu et al., 2018; Barsy et al., 2020) and innervate these disinhibitory cells (Krabbe et al., 2019). Further studies will be needed to elucidate the role of these disinhibitory cells in other amygdala functions.

## GABAergic Projection Neurons

The last group of inhibitory cells present in cortical structures are not, strictly speaking, interneurons, as in addition to often having local axonal collaterals, they give rise to long-range projections to remote brain regions, as it was demonstrated first in the hippocampus (Alonso and Köhler, 1982; Seress and Ribak, 1983). These GABAergic projection neurons typically have large somata and elongated dendrites, often decorated with spines. Many of them are located close to or within the axonal bundles of cortical principal neurons, i.e., in the alveus/stratum oriens of the hippocampus, in the hilus of the dentate gyrus, or in the sixth layer of the neocortex (Tóth and Freund, 1992; Gulyas et al., 2003; Jinno et al., 2007; Tomioka and Rockland, 2007; Takács et al., 2008; He et al., 2016), where they have the largest chance to be innervated in a feedback manner. The most studied GABAergic projection neurons are the hippocampo-septal GABAergic cells that express SST, Calb, NPY in some cases (Tóth and Freund, 1992; Gulyas et al., 2003; Jinno et al., 2007), and interestingly, even VGluT3 (Pelkey et al., 2020). These GABAergic cells receive a high number of excitatory synapses on their membrane surface (~20–35,000) and much fewer inhibitory synapses, many of which originate from the medial septum (~1,000–2,500; Takács et al., 2008), implicating that they are relatively unaffected by interneuronal operation, but are in the position to efficiently monitor the on-going principal neuron activity. Hippocampo-septal inhibitory cells preferentially, if not exclusively target hippocampal interneurons locally (Gulyas et al., 2003), but see (Jinno et al., 2007). Another large group of GABAergic projection neurons expresses muscarinic receptor type 2 (M2; Hajos et al., 1998; Ferraguti et al., 2005). In contrast to hippocampo-septal cells, M2 GABAergic cells can often be found in all layers of the hippocampus and cortex (Hajos et al., 1998; Tomioka and Rockland, 2007), and they prefer to innervate other GABAergic cells (Ferraguti et al., 2005; Katona et al., 2020).

Such GABAergic projection neurons are present also in the amygdala (McDonald et al., 2012; Bienvenu et al., 2015; McDonald and Zaric, 2015). Those GABAergic neurons that project to the basal forebrain (specifically to the substantia innominata and horizontal limb of the diagonal band of Broca, HDB) express SST, Calb, or NPY (McDonald et al., 2012), and nNOS (Vereczki et al., 2021). Interestingly, the majority of these projecting inhibitory neurons are located either paracapsularly, i.e., on the verge of the LA, BA, and BM, often between these nuclei, or in the external capsule (McDonald et al., 2012; Vereczki et al., 2021), which seems to be equivalent with the alveus of the hippocampus, as it is formed by axonal bundles. In addition, GABAegic neurons projecting to the entorhinal cortex have been also identified in the BLA complex (McDonald and Zaric, 2015). These neurons had a similar location and neurochemical profile as those GABAergic cells that send axon collaterals to the basal forebrain and they also contain nNOS (Vereczki et al., 2021). At present, however, it is not clear whether the same GABAergic projection neurons innervate both the basal forebrain and entorhinal cortex, as suggested by their soma localization within the amygdala and their comparable neurochemical profile. M2 GABAergic neurons have also been described in the amygdala (McDonald and Mascagni, 2011). These neurons, similar to those identified in the hippocampus (Hajos et al., 1998), have large somata and long, sparsely ramified dendrites. Some of these neurons are restricted to the external capsule, displaying elongated morphology, whereas others have a multipolar appearance. It has been verified that some of the M2 GABAergic neurons expressing SST project to the entorhinal cortex (McDonald and Zaric, 2015). In general, GABAergic neurons located in the external capsule are strategically positioned to provide both feedback and feedforward inhibition that can even control synaptic plasticity within amygdalar circuits (Morozov et al., 2011).

Those SST GABAergic cells that express high levels of nNOS belong to GABAergic projection neurons (Vereczki et al., 2021), similarly to those neurons that have been described in the hippocampus (Sik et al., 1994; Christenson Wick et al., 2019) and neocortex (He et al., 2016). In the amygdala, the vast majority of strongly immunopositive nNOS GABAergic cells also show immunoreactivity for SST, NPY, and type 1 neurokinin receptor (NK1, substance P receptor; Bocchio et al., 2016). In the LA and BA, 40 and 25% of all SST GABAergic cells, respectively, were strongly immunopositive for nNOS (Vereczki et al., 2021). These nNOS inhibitory cells were located predominantly in the paracapsular zone of the LA and BA, but some of them were present between these two nuclei (Bocchio et al., 2016), resembling the localization of those SST GABAergic neurons that project outside of the amygdala. Spiking of nNOS inhibitory neurons show accommodation and an h-current-mediated sag decorates their voltage responses upon hyperpolarizing current injection (Bocchio et al., 2016), single-cell properties indistinguishable from SST interneurons (Vereczki et al., 2021). Interestingly, these amygdalar nNOS GABAergic neurons were activated during sleep, an effect that may be linked to their serotonergic receptor expression, as 5-HT application changed their tonic firing to bursting mode measured in acute slices (Bocchio et al., 2016). In line with the potential role for nNOS/SST GABAergic neurons in controlling sleep, a recent study has shown that deleting nNOS from cortical SST inhibitory cells led to changes in slow wave sleep as well as in recognition memory (Zielinski et al., 2019). No doubt, specific modulation of the function of nNOS/NPY/SST GABAergic neurons using an intersectional viral strategy is needed in the future to uncover their contribution to sleep and other cortical operations.

GABAergic projection neurons that lack SST immunoreactivity, but express metabotropic glutamate receptor type 1α (mGluR1α), PV, and GABA_A_ receptor subunit α1, have been identified in the rat BLA (Bienvenu et al., 2015). Although the authors of this study named mGluR1α GABAergic neurons as large intercalated cells, because their somata and dendrites were preferentially located within the external capsule, often surrounding intercalated cell masses, these inhibitory neurons may belong rather to the amygdala circuits based on their connectivity. The vast majority of the axons of *in vivo* labeled large mGluR1α GABAergic neurons arborized in the LA and BA, while only a negligible portion of their axon terminals was observed in the central amygdala, and no axons were found in the amygdalo-striatal area, the two striatal structures that are the main projection targets of intercalated cells (Pare and Smith, 1993; Busti et al., 2011; Asede et al., 2015). These large mGluR1α GABAergic cells projected to perirhinal, entorhinal, and endopiriform cortices (Bienvenu et al., 2015). Of note, both locally and remotely, the targets of these GABAergic projection neurons were inhibitory cells, preferentially PV interneurons. Furthermore, mGluR1α GABAergic cells were strongly excited by noxious stimuli (Bienvenu et al., 2015). These data collectively imply that, in addition to VIP interneurons, there may be another GABAergic neuronal element within amygdala circuits that disinhibit principal neurons during the presentation of aversive stimuli, and, therefore, promote associative learning.

In summary, GABAergic long-range projections from the BLA to remote areas originate from various inhibitory cell groups. Undoubtedly, more work is needed to understand their role in circuit function.

## Conclusions

**Figure 3:**
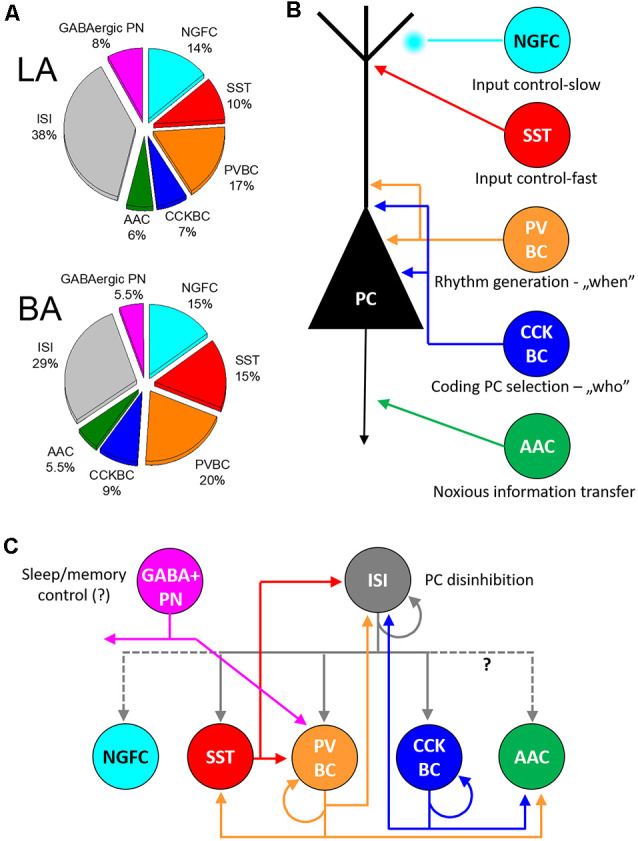
Inhibitory cells, their connectivity, and proposed functions in the circuits of the basolateral amygdala. **(A)** Ratio of distinct types of GABAergic cells in the lateral (LA) and basal amygdala [BA; adopted from (Vereczki et al., 2021)]. **(B)** Five interneuron types innervating the distinct membranedomains of principal cells (PC) may play different roles in circuit operation. **(C)** Connectivity matrix among GABAergic neurons in the basolateral amygdala. Possible functions for GABAergic projection neurons (GABA+ PN) and interneuron-selective interneurons (ISI) are indicated. NGFC, neurogliaform cells expressing NPY; SST, dendrite-innervating interneurons expressing somatostatin; PVBC, parvalbumin-containing basket cells; CCKBC, cholecystokinin-expressing basket cells; AAC, axo-axonic cells.

Recent studies have elucidated that GABAergic cells in the BLA correspond to those inhibitory neurons, both interneurons and projection neurons that were described in other cortical networks based on their morphology, single-cell features, and connectivity (Figures 1, 3). Overall, these results support the view that there are given circuit motifs universally found in all cortical structures (Figure 3). However, there are also notable differences among cortical areas, for instance, the differences in the peak amplitude of postsynaptic responses at the output synapses of perisomatic inhibitory cells relative to each other, a parameter that determines the efficacy of inhibition. Such variabilities along with other inhibitory circuit features likely contribute to distinctive network operations necessary to fulfill the functions of a given cortical region. A better understanding of similarities and differences in microcircuit organizations should help recognize the role of the given cortical area in information processing. To reach this goal, distinct types of interneurons should be manipulated selectively. For instance, at present, PV basket cells and PV axo-axonic cells are impacted simultaneously by using PV-Cre mice, yet it would be ideal to modulate their functions separately, to which end, novel tools and approaches should be developed. In addition, behavioral manipulations using currently available tools like opto- and chemogenetics should be extended to uncover circuit mechanisms as completely as possible, perhaps by combining research efforts of teams, experts of different methodologies. Finally, rigorous and often labor-intense examinations of wiring principles should be implemented to gain deeper insight into network operations at the cellular, synaptic, and microcircuit levels. Combinations of these approaches with high quality and comprehensive investigations will result in a substantial advance in our knowledge about different GABAergic neuron types and their function in the BLA, a prerequisite for understanding the role of distinct inhibitory circuits in normal and pathological amygdala operation.

## Author Contributions

The author wrote this review article.

## Conflict of Interest

The author declares that the research was conducted in the absence of any commercial or financial relationships that could be construed as a potential conflict of interest.
